# Multiple Protein Domains Contribute to Nuclear Import and Cell Toxicity of DUX4, a Candidate Pathogenic Protein for Facioscapulohumeral Muscular Dystrophy

**DOI:** 10.1371/journal.pone.0075614

**Published:** 2013-10-08

**Authors:** Edgardo Daniel Corona, Daniela Jacquelin, Laura Gatica, Alberto Luis Rosa

**Affiliations:** 1 Laboratorio de Biología Celular y Molecular, Fundación Allende, Córdoba, Argentina; 2 Sanatorio Allende, Córdoba, Argentina; Florida State University, United States of America

## Abstract

DUX4 (Double Homeobox Protein 4) is a nuclear transcription factor encoded at each D4Z4 unit of a tandem-repeat array at human chromosome 4q35. DUX4 constitutes a major candidate pathogenic protein for facioscapulohumeral muscular dystrophy (FSHD), the third most common form of inherited myopathy. A low-level expression of DUX4 compromises cell differentiation in myoblasts and its overexpression induces apoptosis in cultured cells and living organisms. In this work we explore potential molecular determinants of DUX4 mediating nuclear import and cell toxicity. Deletion of the hypothetical monopartite nuclear localization sequences RRRR^23^, RRKR^98^ and RRAR^148^ (i.e. NLS1, NLS2 and NLS3, respectively) only partially delocalizes DUX4 from the cell nuclei. Nuclear entrance guided by NLS1, NLS2 and NLS3 does not follow the classical nuclear import pathway mediated by α/β importins. NLS and homeodomain mutants from DUX4 are dramatically less cell-toxic than the wild type molecule, independently of their subcellular localization. A triple ΔNLS1-2-3 deletion mutant is still partially localized in the nuclei, indicating that additional sequences in DUX4 contribute to nuclear import. Deletion of ≥111 amino acids from the C-terminal of DUX4, on a ΔNLS1-2-3 background, almost completely re-localizes DUX4 to the cytoplasm, indicating that the C-ter tail contributes to subcellular trafficking of DUX4. Also, C-terminal deletion mutants of DUX4 on a NLS wild type background are less toxic than wild type DUX4. Results reported here indicate that DUX4 possesses redundant mechanisms to assure nuclear entrance and that its various transcription-factor associated domains play an essential role in cell toxicity.

## Introduction

DUX4 is double-homeodomain transcription factor encoded at the tandem repeat D4Z4 (i.e. FSHD1 locus) on the human chromosomal region 4q35 [1], [2]. D4Z4 repeats belong to a family of human 3.3 kb repeats dispersed through the genome [3], [4]. Shortening of the 4q35-linked D4Z4 tandem repeat [5] is associated with the prevalent form of facioscapulohumeral muscular dystrophy (FSHD, OMIM 158900), the third most common form of inherited myopathy in humans [6]. FSHD1 patients have 1–10 D4Z4 repeat units whereas non-affected individuals have 11–100 D4Z4 repeats [7], [8]. Pathogenic short D4Z4 alleles are hypomethylated and associated with a 4q polymorphic variant called 4qA [9], [10]. FSHD2 patients, who do not have D4Z4 contractions at 4q35, have also decreased DNA methylation at the 4q35 D4Z4-tandem repeat [11].

DUX4 is a nuclear protein endogenously transcribed in myoblasts from FSHD patients [12]. Cultured myoblasts or myotubes from affected individuals express the DUX4 protein in a very limited number of nuclei [13]. The protein is highly expressed in germinal cells in testis [13] and also in cultured pluripotent stem cells derived from fibroblast [13]. The *DUX4* gene is turned off when cultured pluripotent cells are differentiating [13]. Transgene expression of DUX4 in various cultured transfected cells leads to apoptosis [12] and its expression in myoblasts disrupts the normal myogenic regulatory pathway [14], alters normal myotube morphology [14], [15] and increases stress susceptibility [14]. Expression of DUX4 in mice muscles causes a TP53-dependent myopathy, which is dependent on the integrity of its homeodomains [16]. It has been shown that DUX4 homeodomains bind the canonical binding site TAAT [17], [18] and activate the expression of *PITX1*, a gene specifically up-regulated in tissues from FSHD patients [17]. The potential pathogenic role for DUX4 in FSHD [12], [19] is supported by elegant molecular and genetics studies showing that a stable *DUX4* mRNA is transcribed from the distal D4Z4 unit in pathological FSHD alleles [20].

In this work we show that DUX4 has multiple domains driving nuclear import and that its various transcription-factor domains participate in DUX4-mediated cell death. Our results indicate that DUX4 possesses redundant mechanisms to assure nuclear entrance and its transcription factor activity may play a role in FSHD pathogenesis.

## Results

### Three Monopartite NLS Contribute to Nuclear Sorting of DUX4

**Figure 1:**
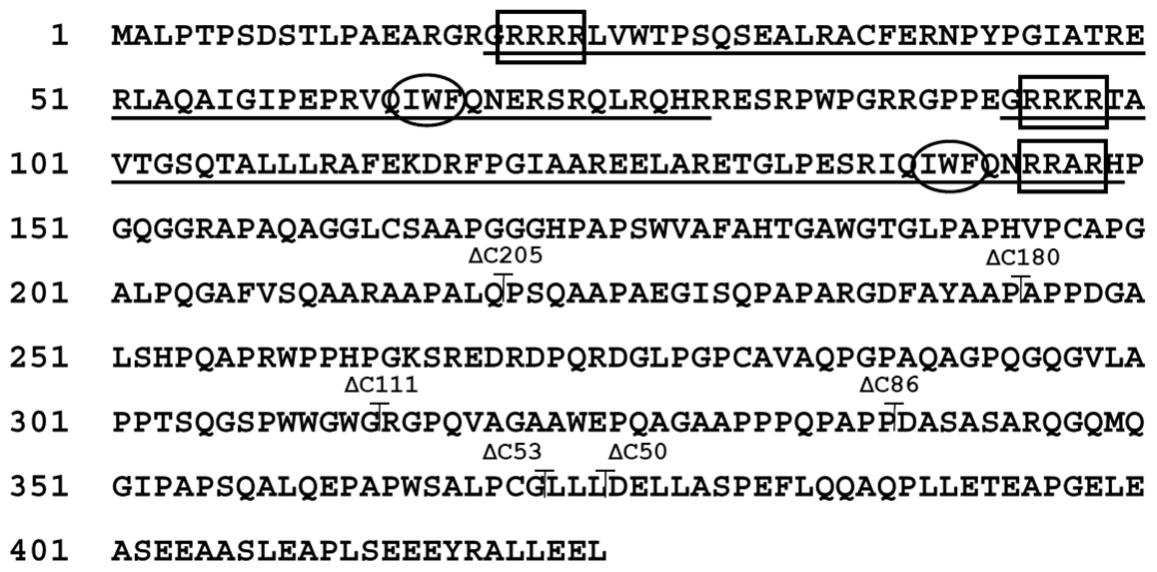
Conceptual DUX4 amino acid sequence. Homeodomains 1 (residues 19 to 79) and 2 (residues 94 to 149) are underlined. NLS1 (RRRR^23^), NLS2 (RRKR^98^) and NLS3 (RRAR^148^) are indicated (*boxes*). IWF1 (IWF^65^) and IWF2 (IWF^140^) are also indicated (*ovals*). The positions of the C-terminal amino acids remaining at the various C-terminal deletion mutants is shown.

Visual and *in silico* (i.e. PSORT II software; http://psort.nibb.ac.jp) inspection of the primary sequence of DUX4 showed the existence of two potential monopartite NLSs: NLS1 (RRRR^23^) and NLS2 (RRKR^98^), located at the N-terminus portion of homeodomains 1 and 2, respectively (Fig. 1) (see Ref. [21]). A less conserved core of basic amino acids (NLS3: RRAR^148^) is present at the C-terminus portion of homeodomain 2 (Fig. 1). The core of basic amino acids at this NLS3 is not conserved in homeodomain 1 (Fig. 1). NLS3 was considered a potential NLS sequence because it matches the consensus (R/K)(R/K)X(R/K), including a C-terminal histidine residue (i.e. RRARH^149^) present in the epidermal growth factor receptor ERB3 (i.e. RRRRH), from the EGFR protein family [22].

**Figure 2:**
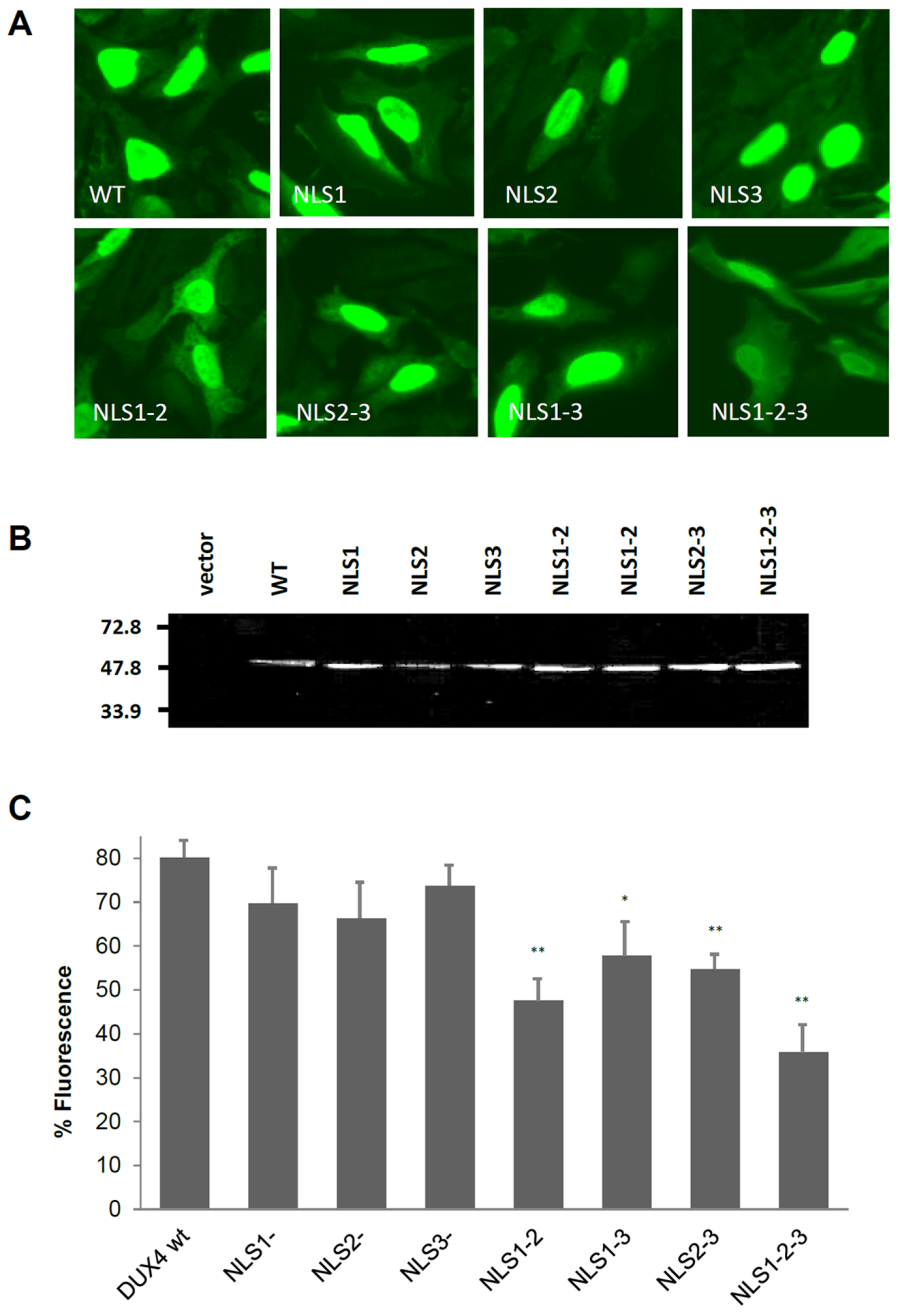
Subcellular distribution of DUX4 ΔNLS mutants. (**A**) DUX4 wild type (*WT*) as well as deletion mutants (*ΔNLS1*, *ΔNLS2*, *ΔNLS3*, *ΔNLS1-2*, *ΔNLS1-3*, *ΔNLS2-3* and *ΔNLS1-2-3*) were expressed in HepG2 cells and immunotsained using the monoclonal mAb9A12 antibody. Non background staining was observed when cells were transfected with the empty pcDNA3.1 vector (not shown; see Material and Methods section). (**B**) Western blot analysis of DUX4 wild type and NLS mutants showed in Fig. 2A, transiently expressed (i.e. 24 hs) in HepG2 cells. Cells transfected with an empty vector are shown (*vector*). The Western blot was developed using mAb9A12. The position of molecular weight markers (i.e. 72.8, 47.8 and 33.9 kDa) is indicated. (**C**) Percentage of nuclear-located DUX4 wild type and NLS mutants as determined by measuring the relative nuclear: cytoplasm fluorescence in HepG2 transfected cells (see Fig. 2A). Data are expressed as mean±SD of two independent experiments. The symbols (**) and (*) indicate significant difference *vs.* DUX4 wt, p<0.01 and p<0.05, respectively. For details, see text.

Site directed mutagenesis was used to prepare single, double and triple ΔNLS deletion mutants of DUX4, lacking the cluster of basic amino acids corresponding to NLS1, NLS2 and/or NLS3 (see Materials and Methods section). To analyze their subcellular location, these DUX4 ΔNLS mutants were expressed in transient transfection experiments using the promoter and enhancer sequences from the CMV (i.e. pcDNA3.1, Invitrogen). To exclude potential artifacts dependent on the massive cell death caused by DUX4 [12], these experiments were performed using short times of transfection (i.e. 24 hr) (see Materials and Methods section). In these studies, transfected HepG2 (Fig. 2A) and HeLa (not shown) cells were immunostained using the anti-DUX4 monoclonal antibody Mab9A12 [17]. Western blot analyses of total protein extracts from these transfected cells indicated that all the DUX4 ΔNLS mutants were properly expressed (Fig. 2B). Figure 2A shows that wild type DUX4 completely localizes to the cellular nuclei [12]. A marked delocalization of DUX4 from nuclei was observed in the triple mutant ΔNLS1-2-3 (Fig. 2A). Partial nuclear delocalization was also observed for the double mutant ΔNLS1-2 and, to a lesser extent, for the double mutants ΔNLS1-3 and ΔNLS2-3. A faint cytoplasmic staining of DUX4 was observed for the single mutants ΔNLS1 and ΔNLS2, suggesting only minor delocalization from nuclei. The single mutant ΔNLS3 mostly localize at the cell nuclei suggesting that it has a minor role in nuclear entrance (Fig. 2A). A quantitative analyses of the subcellular distribution of the various DUX4 NLS mutants is shown in Figure 2C.

Taken together these results indicate that all the analyzed NLSs partially contribute to nuclear entrance, being their apparent relative driving force for nuclear import of DUX4: NLS1 = NLS2>NLS3.

We hypothesized that the ΔNLS1-2-3 mutant still partially localizes to the nuclei because a fraction of DUX4, which is a relatively small molecule (i.e. 50 kDa), may enter the nuclei by passive diffusion [21]. To study this possibility we prepared a fusion of wild type DUX4 to GFP, rendering a large chimeric protein of about 80 kDa (see Materials and Methods section), considered unable to enter the nuclei by passive diffusion [23], [24]. This wild type DUX4-GFP fusion completely localizes to the nuclei (not shown). Also, this fused protein conserves the toxic properties of native wild type DUX4 (see below), indicating that fusion of GFP at the C-terminus of DUX4 does not alter the molecular structure of DUX4 determinants of cell toxicity.

**Figure 3:**
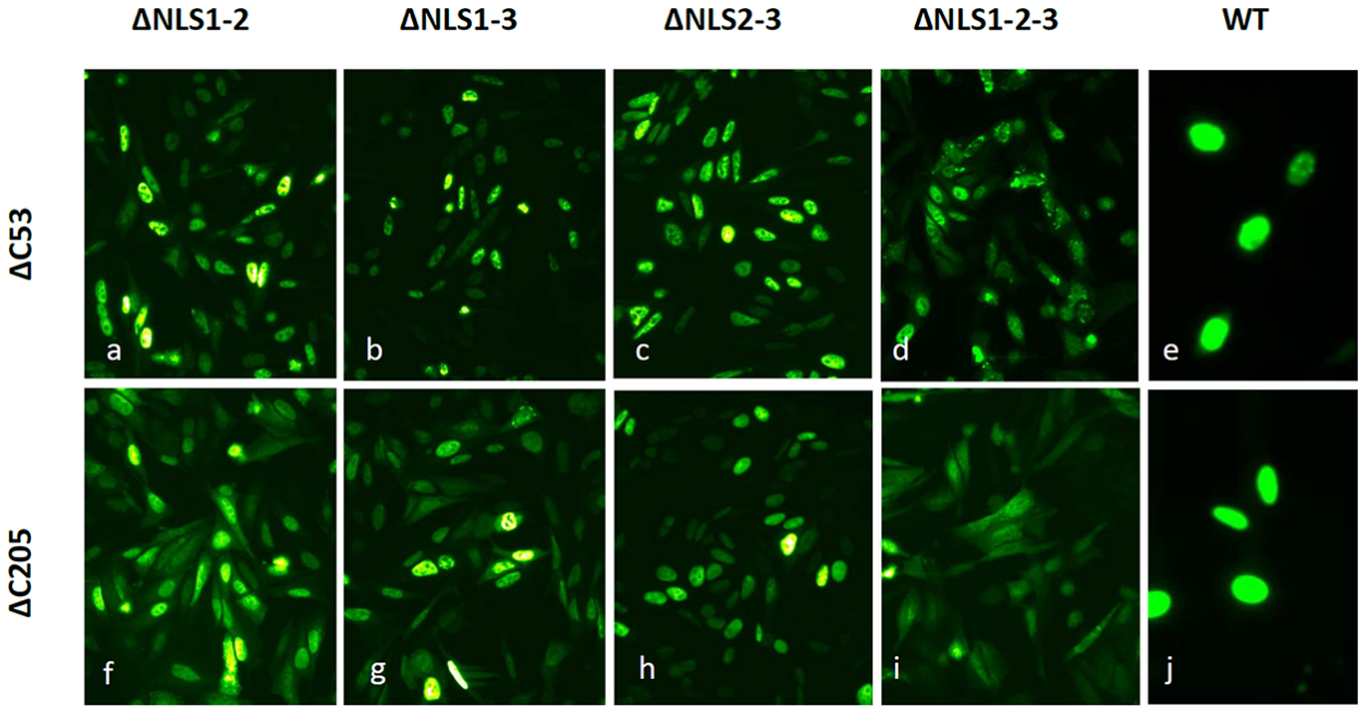
Subcellular distribution of ΔNLS mutants fused to GFP. DUX4 mutants ΔC53 and ΔC205, lacking 53 (*a* to *e*) or 205 (*f* to *j*) amino acid residues from the C-terminus, were used as templates to introduce the double deletions ΔNLS1-2 (*a* and *f*), ΔNLS1-3 (*b* and *g*) and ΔNLS2-3 (*c* and *h*), or the triple deletion ΔNLS1-2-3 (*d* and *i*). Mutants ΔC53 and ΔC205 on a NLS+ background are also shown (*e* and *j*, respectively). All constructs were fused to GFP and expressed in HepG2 cells. Magnifications are 20X (*a* to *d* and *f* to *i*) and 40X (*e* and *j*). For details, see text.

Fusions of DUX4 ΔNLS mutants to GFP (see Materials and Methods section) were constructed using a modified *DUX4* gene carrying a short deletion of 53 amino acids at the C-terminus (see Fig. 1). This DUX4 ΔC53 protein is much less toxic than DUX4 wild type (see below) and does not disturb nuclear localization of DUX4 (Fig. 3*e*). All the fusions to GFP have the expected molecular weight as determined in Western blots developed with a monoclonal antibody against GFP (see below and Materials and Methods section). The ΔNLS-GFP gene fusions have a subcellular distribution (Fig. 3*a* to 3*d*) similar to that observed using the immunostaining approach (Fig. 2A and 2C).

### Nuclear Entrance of DUX4 is not Mediated by α/β Importins

**Figure 4:**
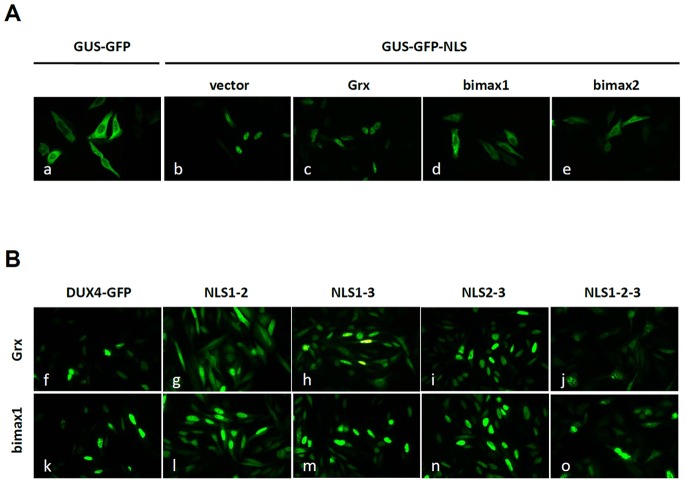
Nuclear entrance of DUX4 is not mediated by α/β importins. (**A**) Transiently transfected GUS-GFP (bacterial β-glucuronidase) is a cytoplasmic protein (*a*) that is imported into nuclei when carrying the NLS from SV40 (GUS-GFP-NLS; see *b*). Co-transfection of GUS-GFP-NLS with a competitive cargo (Grx, yeast glutaredoxin) does not alter its nuclear import (*c*). Co-transfection with plasmids expressing peptides bimax1 (*d*) or bimax2 (*e*) blocks nuclear import of GUS-GFP-NLS. (**B**) GFP fusions of DUX4 wild type (*f* and *k*) or mutants ΔNLS1-2 (*g* and *l*), ΔNLS1-3 (*h* and *m*), ΔNLS2-3 (*i* and *n*), ΔNLS1-2-3 (*j* and *o*) were co-transfected with the control plasmid expressing the competitive cargo Grx (*f* to *j*) or a plasmid expressing bimax 1 (*k* to *o*). Nuclear entrance of DUX4 wild type or mutants was insensitive to the bimax 1 peptide. Similar results were obtained using the bimax 2 (not shown).

Proteins carrying monopartite K(K/R)X(K/R) or bipartite (K/R)(K/R)X10–12(K/R)3/5 (corresponding (K/R)3/5 to at least three of five consecutive lysines or arginines) NLSs [25], [26] are imported into the nucleus via the α/β importins pathway [27], [28]. To study the possibility that NLS1, NLS2 and/or NLS3 transport the DUX4 cargo via α/β importins, we used an experimental strategy based on two recently described nuclear import peptide inhibitors of the α/β importins pathway [29]. These peptides, designed bimax1 and bimax2, bind tightly to α-importin, independently of β-importin, inhibiting the release of the cargo into the nucleus and probably sequestering the α/β-importins into this subcellular compartment [29]. The reporter cytoplasmic protein GUS fused to GFP (i.e. GUS-GFP), as well as a derivative construct containing the NLS from the large antigen T from the virus SV40 (PKKKRKV) (i.e. GUS-GFP-NLS; see Materials and Methods), were used as a control to validate these studies. Fig. 4A shows that GUS-GFP is a cytoplasmic protein which localizes to the nuclei when carrying the NLS^SV40^. Co-transfection of GUS-GFP-NLS with plasmid pGrx1 (i.e. expressing Grx1, a potential competitive cargo; see Materials and Methods section) does not delocalize GUS-GFP-NLS from the nuclei. Thus, co-expression of a cargo containing a *bonafide* NLS does not delocalize GUS-GFP-NLS [29]. Co-transfection of GUS-GFP-NLS with a plasmid expressing bimax1 or bimax 2, however, completely inhibits the nuclear entrance of GUS-GFP-NLS (Fig. 4A). These results validate the use of the bimax peptides to test the functional dependence of DUX4 NLS1, NLS2 and NLS3 on the α/β-importins pathway. Each NLS from DUX4 (i.e. NLS1+, NLS2+ and NLS3+) was independently tested in the corresponding double mutant background (i.e. NLS1+, NLS2+ and NLS3+ were tested in ΔNLS2-3, ΔNLS1-3 and ΔNLS1-2 double mutants, respectively). GFP gene fusions of each double mutant were constructed using a modified *DUX4* gene carrying a deletion of 205 amino acids from the C-terminus (Fig. 1; see Materials and Methods section). This C-terminal region partially contributes to DUX4 nuclear sorting (see below) and may contain a cryptic NLS, potentially covering the results of the bimax peptides inhibition assay. Also, this DUX4-ΔC205 protein is much less toxic than DUX4 wild type (see below) and does not disturb nuclear localization (see Fig. 3*j*). In these studies, NLS1+, NLS2+ and NLS3+ were insensitive to inhibition of the α/β-importins pathway mediated by peptide bimax 1 (see Fig. 4B) or bimax2 (not shown). These experiments indicate that nuclear import of DUX4 mediated by NLS1, NLS2 and NLS3 does not follow the classical nuclear import pathway of α/β-importins. Dependence on the α/β-importins pathway of a potential cryptic NLS present at the C-terminus of DUX4 (see below) was tested using the ΔNLS1-2-3 triple mutant with a wild type C-terminus fused to GFP (see Materials and Methods section). Nuclear import of this protein was not inhibited by the bimax peptides (Fig. 4B *j* and 4B*o*).

### Amino Acids IWF from DUX4 Homeodomains do not Contribute to Nuclear Location

**Figure 5:**
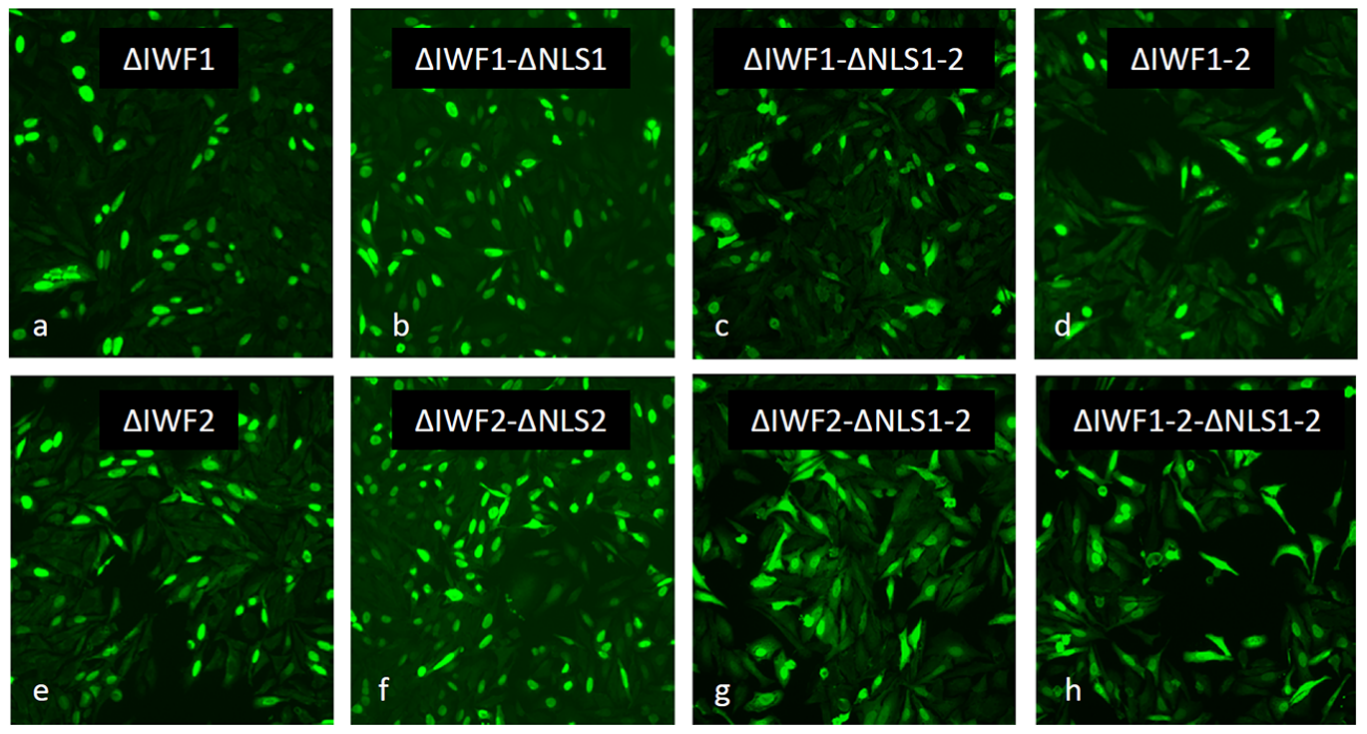
The IWF motif does not contribute to nuclear location of DUX4. Single deletion mutants ΔIWF1 (*a*) and ΔIWF2 (*e*), the double mutant ΔIWF1-2 (*d*), as well as combined deletion mutants ΔIWF1-ΔNLS1 (*b*), ΔIWF2-ΔNLS2 (*f*), ΔIWF1-ΔNLS1-2 (*c*), ΔIWF2-ΔNLS1-2 (*g*) and ΔIWF1-2-ΔNLS1-2 (*h*), were transiently transfected (i.e. 24 hr) into HepG2 and immunostained using the anti-DUX4 monoclonal antibody mAb9A12 (see Materials and Methods section). The single and double ΔIWF mutants completely localize to the nuclei. Combined ΔIWF-ΔNLS mutants localize following the pattern observed for the corresponding ΔNLS mutants. For details, see text.

The IWF sequence is a well conserved motif in homeodomains [30]. This motif is located at the third helix of the homeobox, which participates in protein-nucleic acid and protein-protein interactions [31]. It has been shown that transcription factor TTF1 localizes to the cell nuclei only when it maintains intact its NLS (RRKRR) and its IWF motif [32]. Nuclear import of TTF1 *via* the NLS and nuclear retention through binding to nucleic acids *via* the IWF both appear to contribute to nuclear location of TTF1 [32]. To explore the possibility that IWF sequences from DUX4 homeodomains 1 and/or 2 contribute to nuclear location, and/or nuclear retention of a leaked fraction of DUX4 into the nucleus, we prepared deletion mutants ΔIWF1 (IWF^65^) and ΔIWF2 (IWF^140^). Combined deletion mutants of IWF1, IWF2 and the DUX4 ΔNLSs described above were also prepared (see Materials and Methods section). Cells were transfected with these various mutants and immunostained using the anti-DUX4 monoclonal antibody mAb9A12. Figure 5 shows that single ΔIWF1 and ΔIWF2 mutants, as well as the double mutant ΔIWF1-2, completely localize to the nuclei. Combined ΔIWF and ΔNLS mutants have a subcellular localization that follows the pattern of the corresponding single or combined ΔNLS mutants (compare images from Fig. 5 with Fig. 2A and Fig. 3).

We conclude from these studies that the IWF motifs from homeodomains 1 and 2 do not participate in either nuclear location or nuclear retention of DUX4.

### The C-terminal Tail of DUX4 Participates in Nuclear Import

**Figure 6:**
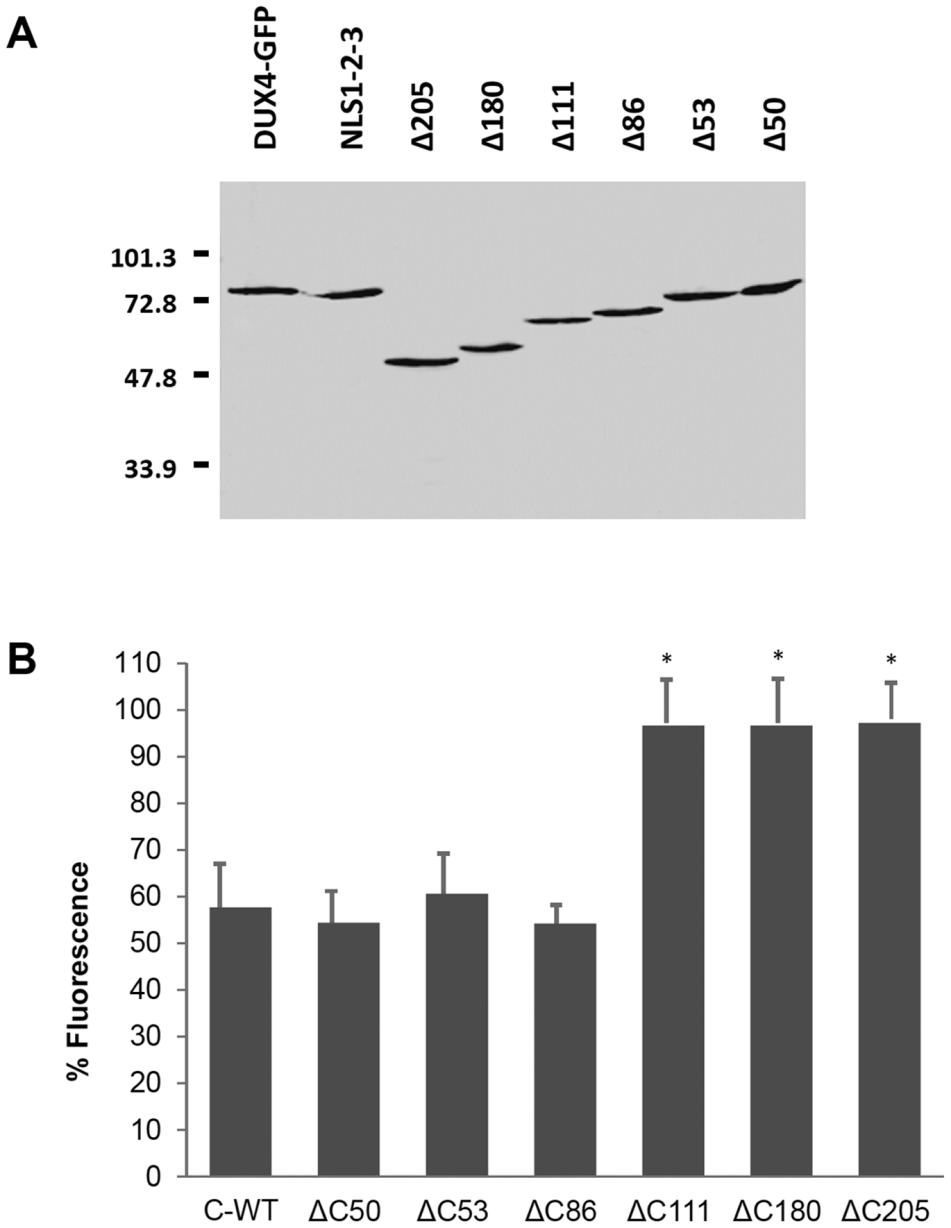
Subcellular trafficking of DUX4 C-terminal deletion mutants. (**A**) Western blot analysis of GFP fusions of DUX4 wild type (*DUX4-GFP*), ΔNLS1-2-3 triple deletion mutant (*NLS1-2-3*) as well as C-terminus deletion mutants ΔC50, ΔC53, ΔC86, ΔC111, ΔC180 and ΔC205 transiently expressed (i.e. 24 hs) in HepG2 cells. All the C-terminus deletion mutants are in a ΔNLS1-2-3 mutant background. The Western blot was developed using a monoclonal anti-GFP antibody. The position of molecular weight markers (i.e. 101.3, 72.8, 47.8 and 33.9 kDa) is indicated). (B) Quantitative analyses of the cytoplasmic distribution of DUX4 C-terminal deletion mutants. HepG2 cells were transfected with plasmids expressing GFP fusions of the triple ΔNLS1-2-3 mutant having either a wild type (*C-WT*) or different deleted (*ΔC50*, *ΔC53*, *ΔC86*, *ΔC111*, *ΔC180* and *ΔC205*) C-terminal domains. The percentage of cytoplasmic green fluorescence was determined as indicated in Material and Methods section. Experiments were performed in blind experiments by counting 20 fluorescent cells from three randomly selected microscope fields. Data are expressed as mean±SD of two independent experiments. The symbol (*) indicates significant difference vs. DUX4 wild type (*C-WT*) (p<0.05).

Results presented above suggest that additional sequences in DUX4 mediate its subcellular trafficking to the nuclei. The potential contribution of the C-terminal region of DUX4 in nuclear sorting was studied using a series of deletion derivatives lacking 50, 53, 86, 111, 180 and 205 amino acids from its C-terminus (see Fig. 1 and Materials and Methods section). To study the role of the C-terminus in nuclear import independently from the contribution of NLS1, NLS2 and NLS3, all the ΔC mutants were prepared in a triple mutant ΔNLS1-2-3 background. Mutants ΔC50, ΔC53, ΔC86, ΔC111, ΔC180 and ΔC205 were fused to GFP and their subcellular localization was analysed in transiently transfected cells. The ΔC-GFP fusion proteins have the expected molecular weight according to Western blots analyses using a monoclonal antibody against GFP (Fig. 6A). Figure 6B shows the quantitative analysis of the nuclei/cytoplasm distribution of the ΔC mutants. As it was shown above, the triple ΔNLS1-2-3 mutant largely delocalizes from the nuclei (Fig. 6B; see also Fig. 2A). Mutants ΔC50, ΔC53 and ΔC86 (see Materials and Methods section) behave similarly to ΔNLS1-2-3 (Fig. 6B, *C* *-WT*), indicating that deletion of a large portion of the C-terminus (i.e. 50, 53 or 86 amino acids) does not modify the nuclear location of DUX4. Mutants ΔC111, ΔC180 and ΔC205, however, almost completely delocalize from the nuclei (Fig. 6B).

Taken together, these results indicate that the C-terminus of DUX4 contributes, independently of the NLSs, to nuclear location of this protein. The domain contributing to nuclear entrance appears to be located around amino acids 314 to 338 (see Fig. 1). We also analyzed the subcellular distribution of a short and large deletion of the C-terminus (i.e. mutants ΔC53 and ΔC205) in a wild type NLS+ background. These mutants completely localize to the nuclei (Fig. 3, *e* and *j*, respectively), suggesting that the monopartite NLS1, NLS2, NLS3 and the C-terminus region around amino acids 314 to 338 constitute independent pathways for DUX4 nuclear entrance (see Discussion). Extensive *in silico* analyses of the region around amino acids 314 to 338 did not show clues on the molecular nature of a potential NLS at this region. Alternatively, DUX4 may constitute a cargo for a homologous or heterologous specifically interacting protein driving DUX4 to the cell nuclei. Perhaps, endogenous expressed DUX4 and/or DUX-like proteins may form heteromeric molecules driving transfected DUX4 into the nucleus.

The finding that the C-terminus region of DUX4 contributes to nuclear entrance offers a potential sensitive strategy to test the differential driving force of the above characterized DUX4 monopartite NLSs. With this aim, we studied GFP-labelled ΔNLS1, ΔNLS2 and ΔNLS3 deletion mutants on the C-terminal deletion background ΔC205. It is assumed that, on this background, sequences NLS1, NLS2 and NLS3 are the only contributing sequences for nuclear import of DUX4. Figure 3 shows that mutant ΔNLS1-2 only partially delocalizes from nuclei in a ΔC53 background (3*a*), is much more delocalized on a ΔC205 background (3*f*). A similar nuclear delocalization was obtained for the double mutants ΔNLS1-3 and ΔNLS2-3 (Fig. 3, compare *b* with *g* and *c* with *h*). Nuclear delocalization was less notorious for the mutant ΔNLS2-3. These results support the contention that the C-terminal domain contributes to the nuclear sorting of DUX4. Also, they confirm that NLS1 and NLS2 are the more relevant NLS recognized in DUX4.

### DUX4-mediated Cell Death Depends on the Integrity of the Homeodomains and the C-terminal Region

**Figure 7:**
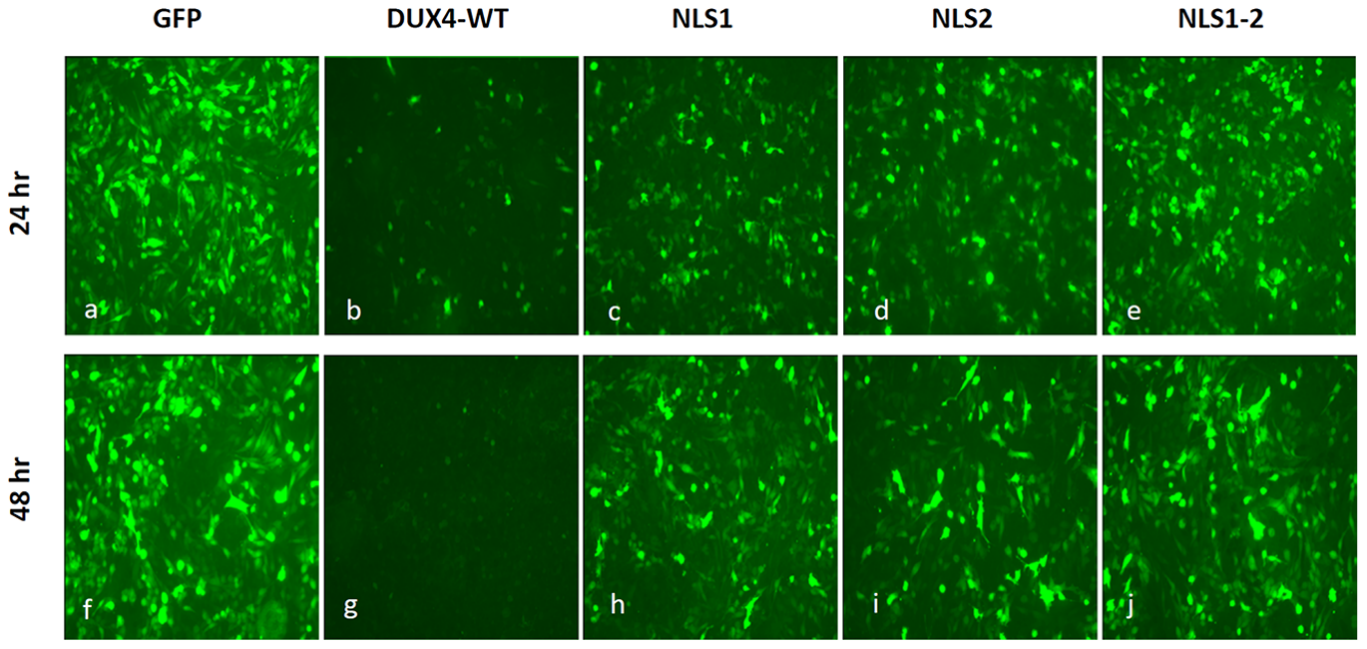
Cell toxicity of DUX4 ΔNLS mutants. A plasmid expressing GFP was co-transfected for 48 (*a* to *e*) or 72 (*f* to *j*) hours with an empty vector (*a* and *f*) or plasmids expressing wild type DUX4 (*b* and *g*) or mutants ΔNLS1 (*c* and *h*), ΔNLS2 (*d* and *i*) and ΔNLS1-2 (*e* and *j*). About 70–80% of green fluorescent cells were observed when a plasmid expressing GFP was co-transfecetd with an empty vector (i.e. *a* and *f*). DUX4-mediated cell death, on the other hand, leaves a very low number of positive fluorescent cells (*b* and *g*) [12]–[19]. A marked reduction in toxicity was observed when using DUX4 mutants ΔNLS1, ΔNLS2 and ΔNLS1-2. For details see text and Materials and Methods section.

**Figure 8:**
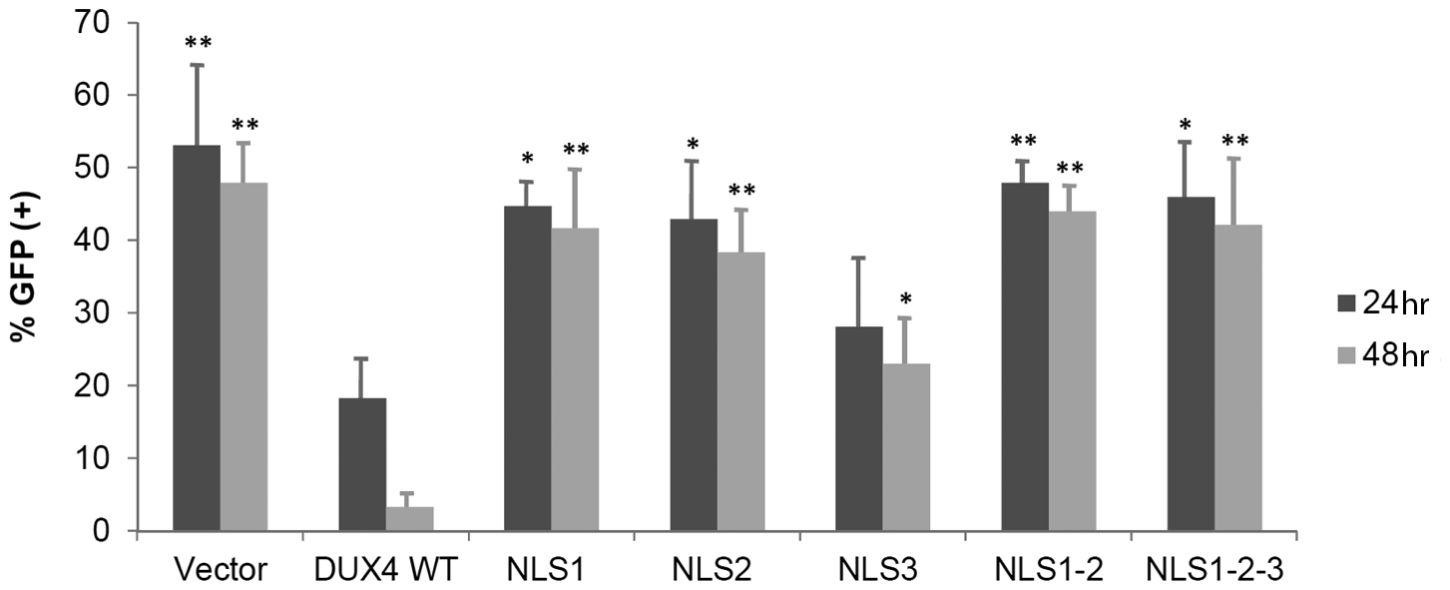
Cell toxicity of DUX4 ΔNLS mutants. The percentage of GFP positive cells was determined in co-transfection experiments at 24 (*dark gray*) or 48 (*light gray*) hours post-transfection (see Materials and Methods section). Scoring was determined in blind experiments by counting 1000–1500 cells (i.e. DAPI staining) from three randomly selected microscope fields. Data are expressed as mean±SD of two independent experiments. The symbols (**) and (*) indicate significant difference *vs.* DUX4 wt, p<0.01 and p<0.05, respectively.

DUX4 is a transcription factor [17], [33], [34] and its normal role requires its homeodomains and the transcriptional enhancer activity associated to its C-terminus [17], [33]. To study these aspects, in a first step we explored if the various characterized DUX4 ΔNLS mutants have different degrees of toxicity. In these experiments we used a co-transfection strategy previously described [12]. This experimental approach uses co-transfection of a *tester* plasmid expressing GFP with a second *testing* plasmid expressing DUX4. The mass ratio tester: testing DNA used for the co-transfecting plasmids was adjusted in a way that most of the cells transfected with the tester plasmid (i.e. expressing *GFP*) are co-transfected with the testing plasmid (see Materials and Methods section) being the observed number of positive GFP cells inversely related to the toxicity of the testing plasmid [12]. Quantitative determination of the percentage of GFP positive cells allows to measure the degree of toxicity of the various DUX4 mutants analyzed. In these studies, duplicated independent experiments were analyzed at 48 and 72 hr following co-transfection. Figure 7 shows that control transfection experiments (i.e. the tester plasmid expressing GFP together with the empty testing vector) have a high number (∼50%) of GFP-positive cells at 48 and 72 hr (*a* and *f*, respectively; see also Fig. 8). A very low number of GFP-positive cells was observed when the wild type version of DUX4 was tested (*b* and *g*), consistent with our original demonstration that DUX4 is a toxic protein and causes cell death when expressed in cultured cells [12]. A dramatic decrease in cell toxicity was observed when cells were transfected with ΔNLS1, ΔNLS2 and ΔNLS1-2 mutants (Fig. 7), being the double mutant ΔNLS1-2 less toxic that the single mutants ΔNLS1 and ΔNLS2 (*e* and *j*). Thus, even when these ΔNLS mutants are mostly localized into the nuclei, like wild type DUX4, its toxic effect is dramatically lower. Fig. 8 shows that single mutants ΔNLS1 and ΔNLS2 have 14% and 21%, respectively, of the toxicity of the wild type DUX4 (see Materials and Methods section) while the double and triple mutants (i.e. ΔNLS1-2 and ΔNLS1-2-3) have 9% and 4%, respectively. On the other hand, the single mutant ΔNLS3 has 56% of the DUX4 wild type toxicity (Fig. 8).

Even when the toxicity of the ΔNLS mutants correlates with their relative presence in the nucleus (i.e. WT>NLS3>NLS2 = NLS1>NLS1-2>NLS1-2-3), it is remarkable that mutants that are still highly concentrated in the nuclei, like the single mutants ΔNLS1 and ΔNLS2, as well as the double mutant ΔNLS1-2, have a low degree of toxicity. These results suggest that DUX4-toxicity is, at least in part, mediated by protein domains that include the NLS sequences. To further explore this idea we incorporated into the various ΔNLS mutants the strong NLS from the T-antigen of virus SV40 (see Materials and Methods section). Transient transfection with these ΔNLS-NLS^SV40^ constructs and immunostaining of DUX4 confirmed that NLS^SV40^ completely re-drives the various ΔNLS mutants to the nuclei (not shown). Analyses of toxicity of these DUX4 ΔNLS-NLS^SV40^ mutants show the same degree of toxicity that the corresponding ΔNLS mutant, irrespective of the presence of NLS^SV40^ (not shown). Thus, the decrease of toxicity of the various DUX4 ΔNLS mutants is not associated with lower nuclear import.

**Figure 9:**
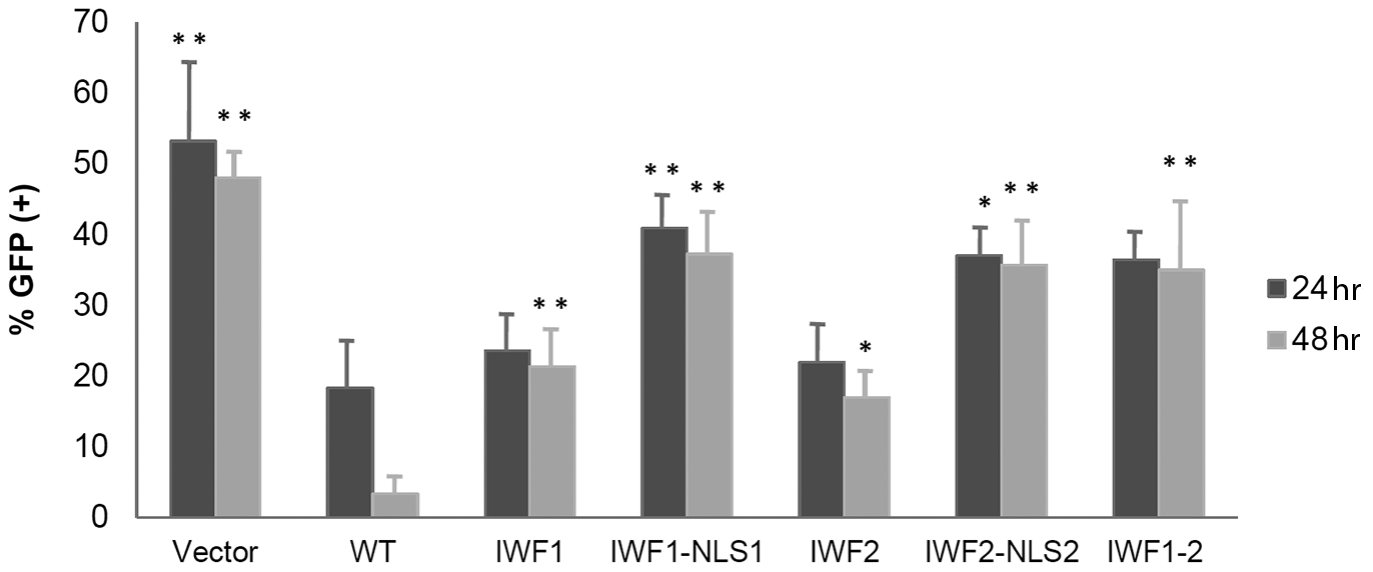
Cell toxicity of ΔIWF mutants. The percentage of GFP positive cells was determined in co-transfection experiments at 24 (*dark gray*) or 48 (*light gray*) hours post-transfection (see Materials and Methods section). Scoring was determined in blind experiments by counting 1000–1500 cells (i.e. DAPI staining) from three randomly selected microscope fields. Data are expressed as mean±SD of two independent experiments. The symbols (**) and (*) indicate significant difference *vs.* DUX4 wt, p<0.01 and p<0.05, respectively.

Considering that the NLS1 and NLS2 sequences are located within the homeodomains (Fig. 1), we explored if homedomain mutants (see Fig. 5) have any effect on cell toxicity. In these studies, single ΔIWF1 and ΔIWF2 mutants, as well as combinations of ΔIWF and ΔNLS mutants, were explored using the GFP co-transfection toxicity assay described above. Figure 9 shows that single ΔIWF mutants are about 40–50% less toxic than the wild type while combinations of the ΔIWF with ΔNLS mutants have a level of toxicity similar to the corresponding ΔNLS mutant. These results suggest that the diminished toxicity of ΔIWF and ΔNLS mutants is based on the alteration of the same molecular determinant of toxicity, perhaps the homeodomains themselves (see Discussion). Supporting this idea, the double mutant ΔIWF1-2 has the lowest level of toxicity, suggesting that both homeodomains independently contribute to the toxic effect of DUX4. In a separate group of experiments we analyzed the contribution of the C-terminal region of DUX4 to cell toxicity. C-terminal deletion mutants ΔC53 and ΔC205 were fused to GFP and used to transiently transfect HepG2 cells (see Materials and Methods section). The percentage of GFP+ cells observed 24 and 48 hr after transfection was scored as an approximate measure of DUX4-mediated cell toxicity. Both C-terminal mutants ΔC53 and ΔC205 were dramatically less toxic than the wild type protein fused to GFP (not shown).

## Discussion

DUX4 is a nuclear, endogenously expressed protein [13]. Low-level expression of DUX4 compromises cell differentiation in myoblasts [14] while its overexpression induces apoptosis in cultured cells [12], a phenomenon which appears to involve p53 activity [16]. DUX4-mediated cell death is a ubiquitous phenomenon occurring in many cell types and living organisms [14], [19]. The finding that DUX4 mRNA is stably expressed in myoblasts only from pathogenic FSHD haplotypes [20] supported its potential pathogenic role in FSHD [17], [20], [35], [36]. Stabilization of the DUX4 transcript is mediated by a poly(A) signal present only at permissive pathological FSHD alleles [20]. It is unknown why high level expression of DUX4 in testes is not toxic [13]. Perhaps the normal function of DUX4 is associated with the co-expression of a tissue-specific, constitutive or developmentally-regulated protein that blocks or bypasses its toxic effect.

DUX4 is a transcription factor evolutionarily conserved in several species [4], [17], [33], [34]. The normal function of DUX4 may require nuclear entrance as well as the integrity of its homeodomains and its acidic C-terminal tail [37], [38]. The N-terminal ends of DUX4 homeodomains have been considered responsible for subcellular trafficking of DUX4 to the nuclei [21]. Nuclear sorting of proteins depends on NLSs, generally consisting of clusters of basic amino acids [39]. Model monopartite and bipartite sequences are represented by the NLS from the large T antigen of virus SV40 (PKKKRKV^132^) [25] and the NLS from nucleoplasmin (KRPAATKKAGQAKKKK ^170^) [26], respectively. In this work we determined that DUX4 sequences NLS1 and NLS2, at the N-terminal ends of the homeodomains, only partially contribute to nuclear entrance. Mutagenesis and deletion analyses indicate that additional sequences (i.e. NLS3) as well as the C-terminal domain of DUX4 contribute to nuclear sorting. Functional redundancy was observed for the various DUX4 NLSs: single NLS mutants only partially delocalize from nuclei. Loss of nuclear localization was more important for the double (ΔNLS1-2, ΔNLS1-3, ΔNLS2-3) and triple (ΔNLS1-2-3) mutants. The existence of additional molecular determinants of nuclear entrance in DUX4 was indicated from the fact that the triple ΔNLS1-2-3 mutant still partially localizes in nuclei. Analyses of various C-terminus deletion derivates of DUX4, in a mutant background ΔNLS1-2-3, indicated that a short C-terminal sequence, around amino acids 314 and 338, participates in DUX4 nuclear entrance. Thus, multiple protein domains from DUX4 contribute to subcellular trafficking of this protein.

Protein containing classic NLSs are imported to the nucleus by a heteromeric protein complex composed of importin α and importin β [27], [28]. In this work we used the peptides called “bimax”, powerful inhibitors of the nuclear import pathway [29], to explore if the various NLS recognized in DUX4 enter the nuclei using the α/β importin pathway. Validation of the experimental strategy was performed using a GUS-based reporter protein containing NLS^SV40^. An independent molecular analysis of each DUX4 NLS showed that none of these sequences drives the protein to the nuclei *via* the α/β importin pathway.

Homeodomains are formed by three α-helices and a flexible N-terminal arm [40], [41]. The third helix, also known as the recognition helix, specifically interacts with the major groove of DNA, while the N-terminal arm interacts with the minor groove [42]. Key amino acids at these regions are IWF and Q^“50”^ [30], [43]. In this work we studied the contribution of DUX4 homeodomains to both subcellular traffic and toxicity of DUX4. Single deletion of DUX4 IWF1 and IWF2 sequences, as well as a double deletion IWF1-IWF2, does not affect the subcellular location of DUX4. Thus, loss of IWF sequences, potentially determining DUX4 binding [17] to DNA and/or retention of DUX4 at the nuclei [32], does not modify DUX4 nuclear location. The IWF mutants have a marked reduction of DUX4 toxicity similar to that observed for the various DUX4 ΔNLSs mutants. NLS1 and NLS2 mutants were also less toxic when carrying the sequence NLS^SV40^ which completely re-drives these mutants to the nuclei. Thus, the low toxicity of DUX4 ΔNLS mutants would be explained because NLS1 and NLS2 partially overlap, or are immediately adjacent, to the nuclei acid binding region of DUX4 [44]. It is known that basic amino acids from the N-terminus of homeodomains directly interact with the DNA-minor groove [30], [42] and disruption of these sequences may affect the DNA-binding activity of DUX4 and/or its activity as a transcription factor. Less toxic variants of DUX4 were also obtained when deleting the C-terminal region of the protein. This C-terminal domain of DUX4 has the signature of a transcription factor and differs from the non-toxic DUX4 highly homologous protein DUX4c [34].

Results presented in this work suggest that DUX4 mediates its toxic effect by: 1) the binding of DUX4 to physiological and/or non-physiological target(s) *via* both homeodomains [17], and 2) recruiting additional molecules *via* its C-terminus as a transcription factor [33]. DUX4 expressed in myoblasts may compete for specific target binding sites and cofactors participating in myotube differentiation to disrupt a normal progression of this pathway (see Ref. [14]). Overexpression of DUX4 in various cultured cell models and organisms may lead to apoptosis via a non-physiological pathway dependent on aberrant higher cellular amounts of DUX4.

Shortening of the 4q35 region associated to FSHD and characterization of the D4Z4 repetitive unit were published in 1993 and 1994, respectively [1], [5]. DUX4 has emerged as the most attractive candidate pathogenic protein in FSHD [20], [35], [36]. Studies directed towards an understanding of the normal biological role of DUX4 as well as its molecular connection with the pathophysiology underlying FSHD are in progress. Results reported here are relevant to the biology of DUX4 and could have an immediate impact on the basic knowledge and potential pathogenic role of DUX4 in FSHD, as well as on the future rational therapeutic approaches to cure FSHD.

## Materials and Methods

### DNA Manipulations

A vector expressing the DUX4 gene was constructed by subcloning a 1.517 bp *EagI*/*KpnI* fragment, obtained from plasmid pGEM/42 [12], into the *NotI/KpnI* sites of pcDNA3.1 (Invitrogen). ΔNLS mutants were generated using the procedure described on the QuikChange® II Site-Directed Mutagenesis kit (Stratagene) as follows: methylated template plasmid DNA was purified from *E. coli* XL1-Blue (dam+). Reaction conditions for mutagenesis were 1.0 mM MgCl2, 2.0 mM of each dNTP, 125 ng of each reverse and forward primers, 20 ng of template DNA and 2.5 U of Pfx polymerase (Invitrogene) using a final volume of 50 ul. DNA was denatured during 30 seconds at 94°C and PCR was performed using 16 cycles of 30 seconds at 94°C, 1 min at 55°C and 7 min at 68°C. PCR products were digested with *Dpn*I to eliminate the methylated template DNA and used to transform competent XL1-Blue. Primers used for mutagenesis are shown in Table 1. The NLS from the T-antigen of virus SV40 (NLS^SV40^) was introduced at the N-terminus of DUX4 ΔNLS mutants by directional cloning. Briefly: a double-stranded oligonucleotide encoding a start codon (ATG) followed by the NLS^SV40^ (PKKKRKV) (see Table 1) was digested with *XbaI* and *XhoI* and cloned directionally into the *XbaI* and *XhoI* sites present at the 5′ of DUX4. All the mutant constructions were verified by DNA sequencing.

**Table 1:** Primers used for mutagenesis.

Name	Sequence (5′ to 3′)	Study
NLS1-F	GAAGCCCGGGGACGAGGACTCGTTTGGACCC	Deletion NLS1 (forward)
NLS1-R	TCCTCGTCCCCGGGCTTCCGCGGGGAGGGTG	Deletion NLS1 (reverse)
NLS2-F	CGCGGCCCGCCAGAAGGCACCGCCGTCACCG	Deletion NLS2 (forward)
NLS2-R	GCCTTCTGGCGGGCCGCGTCTCCCGGGCCAG	Deletion NLS2 (reverse)
NLS3-F	GATTCAGATCTGGTTTCAGAATCACCCGGGACAG	Deletion NLS3 (forward)
NLS3-R	CTGTCCCGGGTGATTCTGAAACCAGATCTGAATC	Deletion NLS3 (reverse)
H1IWF-F	GAGCCCAGGGTCCAGCAGAATGAGAGGTCA	Deletion IWF1 (forward)
H1IWF-R	TGACCTCTCATTCTGCTGGACCCTGGGCTC	Deletion IWF1 (reverse)
H2IWF-F	GGAGTCCAGGATTCAGCAGAATCGAAGGGCCA	Deletion IWF2 (forward)
H2IWF-R	TGGCCCTTCGATTCTGCTGAATCCTGGACTCC	Deletion IWF2 (reverse)
UNI-F	TAT*GCTAGC*CGATGGCCCTCCCGACACCCT	GFP fusion (forward)
DUX4-R	AA*GGTACC*ATAAGCTCCTCCAGCAGAGCCC	GFP fusion (reverse)
ΔC180-R	AA*GGTACC*ATCGGGGCGGCGTAGGCGAAATC	GFP fusion (reverse)
ΔC112-R	AA*GGTACC*ATGCCCCAGCCCCACCACGGACTC	GFP fusion (reverse)
ΔC88-R	AA*GGTACC*ATGGGCGCGGGCTGGGGAGGTG	GFP fusion (reverse)
ΔC53-R	AA*GGTACC*ATCAGCAGCAGGCCGCAGGGGAGTG	GFP fusion (reverse)
SV40-F	ATTCTAGAGCCACCATGGCGCCGAAGAAGAAGCGGAAGGTCCTCGAGCG	Cloning NLS^SV40^ (forward)
SV40-R	CGCTCGAGGACCTTCCGCTTCTTCTTCGGCGCCATGGTGGCTCTAGAAT	Cloning NLS^SV40^ (reverse)

### GFP Gene Fusions

Plasmid pEGFP-N1 (Clontech, Palo Alto, CA) was used to clone EGFP at the C-terminus of the various DUX4 mutants. Fusions to wild type DUX4 and to deletions mutants ΔNLS1-2-3, ΔC180, ΔC111, ΔC87 and ΔC50 were prepared by subcloning into pEGFP-N1 the corresponding fragments obtained by PCR from the various mutants prepared in plasmid pcDNA3.1. PCR reactions contained a universal forward primer (UNI-F; Table 1), having the DUX4 start codon (ATG), and a specific reverse primer (see table 1). PCR products were digested with *NheI* (restriction site on primer UNI-F) and *KpnI* (restriction site on the reverse primer) and cloned directionally into pEGFP-N1. Gene fusions to GFP on backgrounds ΔC205 or ΔC54 were prepared as follows: plasmid DNA from mutants ΔNLS1, ΔNLS2, ΔNLS3, ΔNLS1-2, ΔNLS1-3, ΔNLS2-3 and ΔNLS1-2-3 was first digested with *XhoI* followed by partial digestion with *PstI*. DNA fragments of 682 bp and 1.180 bp, corresponding to ΔC205 and ΔC53, respectively, were purified from agarose gels and subcloned in-frame at the N-terminus of GFP using sites *XhoI* and *PstI* from pEGFP-N1. All constructs were verified by DNA sequencing.

### Cell Culture and Cell Transfection

The subcellular distribution of DUX4 mutants was analyzed using transiently transfected HepG2 (human hepatic carcinoma; ATCC HB8065) and HeLa cells. In these studies, cells were grown to 80–90% of confluence in RPMI 1640 plus 10% (v/v) fetal bovine serum and appropriate supplements and transfected using Lipofectamine 2000 (Invitrogen). Endogenous expression of DUX4 was not detected in these cells. Immunocytochemical staining was performed using anti-DUX4 monoclonal antibodies Mab9A12 [17]. Transfected cells were washed three times with PBS and then fixed in 4% paraformaldehyde/sucrose for 25 min at room temperature. Cells were permeabilized with methanol, 15 min at −20°C, followed by 5 min at room temperature. After incubation with PBS/5% BSA for 45 min the cells were incubated at 4°C overnight with the primary antibody diluted 1∶40 in PBS/1% BSA. The next day, cells were washed three times with PBS and incubated with the secondary antibody. Slides were mounted using FluorSave (Calbiochem, La Jolla, CA) and fluorescence images were captured under a Zeiss Axioplan-2 fluorescence microscope. Quantitative determination of the distribution of DUX4 at the nuclei and cytoplasm subcellular compartments was performed using the ImageJ software and digital images of DUX4 transfected cells immunostained with the monoclonal antibody Mab9A12 (see Fig. 2a and 2b).

### Western Blot Analysis

Transfected cells were also analyzed by Western blot. Cells were harvested in RIPA-DOC buffer (150 mM NaCl, 1% Triton X-100, 1% Na-deoxycholate, 0.1% SDS, 50 mM Tris-HCl, pH 7.2) supplemented with a cocktail of protease inhibitors (SIGMA, Catalogue number P8340). Cell lysates were clarified by centrifugation and extracted proteins boiled in Laemmli’s buffer for 10 min. After electrophoresis on 12% SDS-PAGE proteins were electroblotted into PVDF filters (PolyScreen) using a TransBlot cell (BioRad). Membranes were blocked in 5% nonfat dry milk in TBST (20 mM Tris-HCl, pH 7.5; 150 mM NaCl, 0.1% Tween 20) at 4°C overnight and subsequently incubated with the primary antibody at 4°C diluted in 1% nonfat dry milk – TBST. After three washings with TBST, membranes were incubated with anti-mouse IgG (diluted 1∶20000) coupled with infrared dyes (IRDye700 and IRDye800). Blots were scanned using Odyssey Infrared Imager (LI-COR Biosciences, UK).

### bimax 1 and Bimax 2 Peptides

The use of bimax 1 and 2 peptides was performed as described [29]. Briefly: HepG2 cells were co-transfected with 300 ng of plasmid bimax 1, bimax 2 (not shown) or pGRX1 (i.e. expressing the nuclear protein Grx1) [29] and 700 ng of either GUS-GFP, GUS-GFP-NLS^SV40^, double mutants ΔNLS1-2, ΔNLS1-3 or ΔNLS2-3 fused to GFP in a ΔC205 background, DUX4 wild type or ΔNLS1-2-3, fused to GFP using 1.5 ul of lipofectamine 2000 (Invitrogen) as specified by the manufacturer. Subcellular distribution of green fluorescence was determined under the microscope.

### Cell Toxicity Assays

The effect of the ΔNLS and ΔIWF mutations on the DUX4-mediated cell death was studied using a GFP-based co-transfection assay previously developed in our laboratory [12]. Briefly, HepG2 cells were co-transfected with 150 ng of pEGFP-N1 and 350 ng of the various analyzed constructs or the empty pcDNA3.1(+) vector (500 ng total DNA) using 0.75 ul of Lipofectamine 2000 (Invitrogen) as specified by the manufacturer. The total amount of DNA used (500 ng) was in the linear range of response between amounts of DNA and number of transfected cells. Duplicated independent transfection and co-transfection experiments were analyzed at 24 and 48 hours. The percentage of cells expressing GFP was determined on random selected images obtained at the fluorescence microscope. About 1,500–2,000 cells were examined [i.e. positive DAPI (4′,6-diamidino-2-phenylindole) staining]. Results were expressed as percentage of GFP positive cells ± SD.

### Statistical Analysis

Data in Figures 2C, 6B, 8 and 9 are expressed as mean±SD. Statistical differences were determined by one-way ANOVA with Dunnett’s post test using GraphPad InStat v.3.0 software.
